# High-Phosphate-Stimulated Macrophage-Derived Exosomes Promote Vascular Calcification via let-7b-5p/TGFBR1 Axis in Chronic Kidney Disease

**DOI:** 10.3390/cells12010161

**Published:** 2022-12-30

**Authors:** Qing Li, Cailin Zhang, Jia Shi, Yi Yang, Xue Xing, Yanan Wang, Xiaona Zhan, Le Wang, Gang Xu, Fan He

**Affiliations:** Department of Nephrology, Tongji Hospital, Tongji Medical College, Huazhong University of Science and Technology, Wuhan 430030, China

**Keywords:** vascular calcification, macrophage, exosome, CKD, TGFBR1, let-7b-5p

## Abstract

Although macrophage infiltration has been proven to increase calcified artery media in chronic kidney disease (CKD) patients, the mechanism by which macrophages are involved in vascular calcification (VC) progression remains unclear. Taking advantage of miRNA-seq, RNA-seq, dual-luciferase reporter assay, qRT-PCR, and arteries from CKD patients as well as CKD mouse models, we identified that high-phosphate-stimulated macrophage-derived exosomes (Mexo-P) suppressed let-7b-5p expression in VSMCs, which further upregulated TGFBR1. Moreover, gain-and-loss-of-function assays were used to determine the regulatory effects and downstream mechanism of let-7b-5p and TGFBR1 on VC. Mechanically, Mexo-P induced VSMC TGFBR1 upregulation by suppressing let-7b-5p, which further amplifies SMAD3/RUNX2 signaling and thereby contributes to VC. Our findings indicate that macrophage-derived exosomes promote CKD-associated VC through the let-7b-5p/TGFBR1 axis in high-phosphate conditions. Our study provides insight into macrophages associated with VC, which might be potential therapeutical targets for VC.

## 1. Introduction

Vascular calcification (VC) is a strong prognostic marker of all-cause and cardiovascular mortality in chronic kidney disease (CKD) patients [1,2]. It occurs in approximately 27–40% of CKD patients [3]. VC can occur during any stage of CKD and often becomes progressively more severe with the development of CKD [3]. According to the location of calcium accumulation, VC can be classified into different histopathological types: VC in intima, VC in media, and cardiac valve calcification [4]. VC in media is the most predominant, accounting for over 50% of the VC in CKD patients [3]. Numerous lines of evidence suggest that vascular calcification is an active process and that vascular smooth muscle cell (VSMC) osteogenic transdifferentiation is an important mechanism involved [5]. Many factors, such as phosphate, calcium, and indoxyl sulfate, are involved in the regulation of VSMC osteogenic transdifferentiation [6]. Moreover, many previous studies have illustrated the mechanism of VSMC osteogenic transdifferentiation, such as the impact of RUNX2, BMP7, and pro-inflammatory factors [5]. Macrophages have been intensively studied in intimal calcification [7]. They play an important regulatory role in the occurrence, development, and regression of intimal calcification [7,8]. However, there are fewer studies of macrophages in medial calcification.

Previous studies have shown that macrophages infiltrate into the media of calcified arteries [8], suggesting the potential regulatory role of macrophages for VC; however, little is known about the impact and corresponding mechanisms of infiltrating macrophages in VC. Our work has also identified the infiltration of macrophages into media, especially in CKD patients with higher coronary artery calcification (CAC) scores.

Exosomes are extracellular nanometer-sized (40–100 nm) vesicles [9]. They are thought to be generated by all cells [10]. Exosomes are known to be involved in cell-to-cell communication in health and disease, including cardiovascular systems [11]. They contain numerous cargo molecules, including mRNAs, microRNAs, long non-coding RNAs (lncRNAs), proteins, and lipids that participate in intercellular signaling [9,11,12]. Therefore, exosomes are widely studied for their potential to act as biomarkers and therapeutic agents, including in the cardiovascular system [11]. Multiple groups have reported that exosomes secreted from activated monocytes or macrophages transmit pro-inflammatory signals through several distinct mechanisms [12,13,14]. Furthermore, macrophage-derived exosomes were found to affect VSMC transdifferentiation in atherosclerosis [15,16] and vascular injury [17,18] through non-coding RNA or other cargo. However, the precise function of macrophages and the impact of their non-coding RNA cargos remain poorly understood.

Our present study investigated the potential promotional effect of high-phosphate-stimulated macrophage-derived exosomes in VC and identified let-7b-5p/TGFBR1 as the pathway regulating VSMC calcified phenotype transdifferentiation, which provides novel insight into the VC process and potential therapeutic targets for VC.

## 2. Methods and Materials

### 2.1. Patient Artery Samples

A cohort of 21 patients (12 males) with end-stage renal diseases undergoing arteriovenous fistula operation was enrolled at Tongji Hospital from January to December 2021. The baseline demographics and clinical characteristics of CKD patients are shown in Table S1. For the use of human plasma and artery tissue specimens, patient consent was obtained as approved by the Ethics Committee of Tongji Medical College, Huazhong University of Science and Technology (TJ-IRB20211119). The investigations conform to the principles outlined in the Declaration of Helsinki.

The coronary arterial calcification (CAC) score calculation was performed based on chest computed tomography (CT) quantified based on CT using the Agatston score following the protocol described in Tang Z. et al.’s work [16]. Patients were divided into three groups based on CAC scores: mildly calcified (CAC 0–99 scores), moderately calcified (CAC 100–399 scores), and severely calcified (CAC > 400 scores).

### 2.2. Cell Culture and Treatments

The mouse vascular smooth muscle (MOVAS) cell line (CRL-2797TM) and human aortic smooth muscle cell (HASMC) strain (PCS-100-012TM) were from American Type Culture Collection (ATCC, Rockville, MD, USA). The human monocytic cell line THP-1 (TIB-202™) was also from ATCC and was kindly offered by Dr. X. Liu (Neurosurgery Department, Tongji Hospital). For cell culture and treatment details, please see the Supplementary Materials.

### 2.3. Animal Models

Adenine-induced mice CKD models were used in this study, and a high-phosphorus diet was adapted to induce calcification. First, 8-week-old male C57BL/6 mice were treated with adenine (200 mg/kg) via gavage administration daily and fed with a 1.8% phosphorus diet for 5 weeks. The animals were anesthetized i.p. with 90 mg/kg ketamine and 10 mg/kg xylazine, and anesthesia depth was checked by toe pinch. Blood was drawn via the heart and the killing of the animals was achieved by cervical dislocation under deep anesthesia (Figure S1). Animal experiments were approved by Animal Care and Ethics Guidelines (HZAUMO-2021-0152) and performed according to the guidelines from Directive 2010/63/EU of the European Parliament on the protection of animals used for scientific purposes or the NIH Guide for the Care and Use of Laboratory Animals.

### 2.4. Statistical Analysis

Statistical analysis was conducted using GraphPad Prism version 8.0 software (Graphpad software, San Diego, CA, USA). If normally distributed, values for continuous variables with normal distribution are provided as means (standard deviation). Otherwise, they are provided as medians (interquartile range). A one-way analysis of variance, unpaired *t*-test, Mann–Whitney U test, or Kruskal–Wallis test was used for continuous data as appropriate. Chi-square tests or Fisher’s exact tests were used for categorical variables. The number of samples for each piece of data (n) is mentioned in the figure legends. The statistical significance is expressed as follows: * *p* < 0.05; ** *p* < 0.01; *** *p* < 0.001; **** *p* < 0.0001; and n.s., not significant.

For more details concerning the reagents, chemicals, and antibodies used; aortic rings culture; calcification analysis; miRNA intervention; qRT-PCR; knockdown and overexpression of TGFBR1; immunohistochemistry; Western blot assays; exosome isolation and validation; transcriptome and miRNA sequencing analysis and processed data; plasmid constructs; and reporter assay, please see the Supplementary Materials. The primers used in the study are shown in Table S2.

## 3. Results

### 3.1. Increased Macrophage Infiltration in Media of CKD Patients with Aggravated Calcification and Increased Blood Phosphate

**Figure 1:**
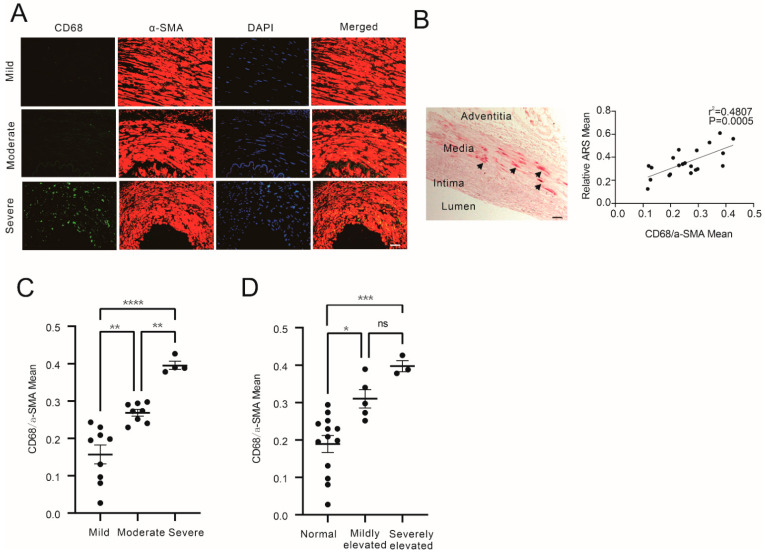
Macrophage infiltration increased in the media of CKD patients with aggravated calcification and increased blood phosphate. (**A**) Representative IF staining of CD68 and α-SMA of arteries of mildly, moderately, and severely calcified CKD patients. (**B**) Left, representative ARS staining of artery of patients (arrows show positive ARS staining); right, correlation of fluorescence intensity mean of CD68 normalized to α-SMA positive area (CD68/α-SMA) and mean area of positive ARS Staining in arteries from CKD patients. (**C**) Comparison of relative CD68 fluorescence intensity in the media between CKD patients with different degrees of artery calcification (one-way ANOVA, n > 3). (**D**) Comparison of relative CD68 fluorescence intensity in the media between CKD patients with different blood phosphate levels (normal, mildly elevated, severely elevated) (one-way ANOVA, n > 3). * *p* < 0.05, ** *p* < 0.01, *** *p* < 0.001, **** *p* < 0.0001, n.s. not significant. Scale bar, 40 μM.

To identify the macrophages in media calcification, immunofluorescence staining of CD68 was performed with radial arterial tissues from CKD patients (Table S1) with different levels of vascular calcification according to CAC score. As shown in Figure 1A, the infiltration of macrophages was increased in the severely calcified group compared to the mildly or moderately calcified group. Alizarin Red S (ARS) staining was also performed to measure local calcification levels. The semi-quantification of mean ARS staining positive area and semi-quantification of macrophage IF intensity were found to be positively correlated (Figure 1B, r^2^ = 0.4807, *p* = 0.0005). The macrophage infiltration in media was also found to be elevated in the arterial tissues of CKD patients with aggravated calcification (Figure 1C) and elevated blood phosphate (Figure 1D).

### 3.2. High-Phosphate-Stimulated Macrophages Releasing Exosomes Promotes Vascular Calcification

**Figure 2:**
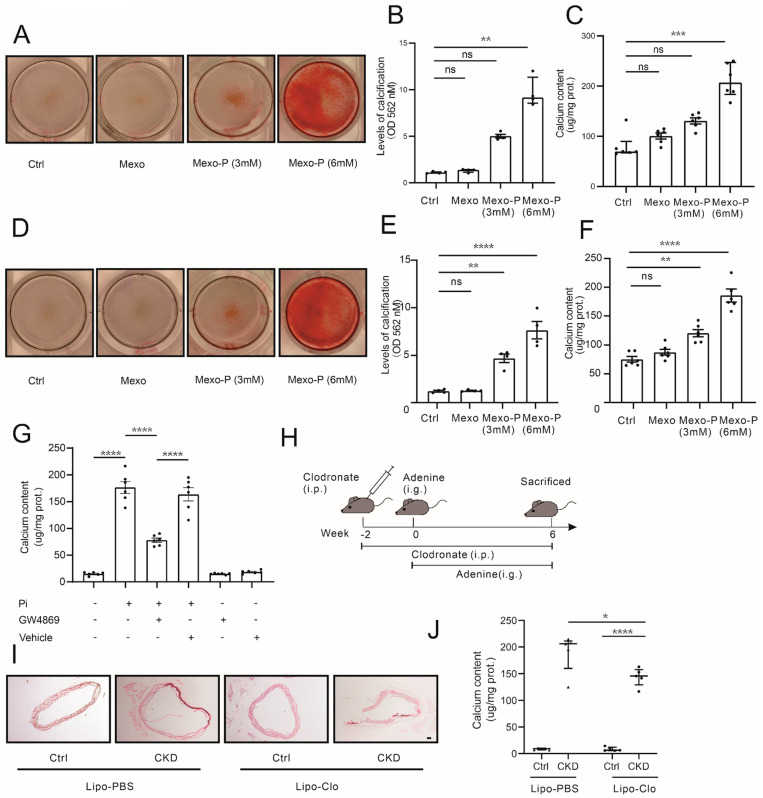
High-phosphate-stimulated macrophages releasing exosomes promote vascular calcification. Representative images of ARS staining (**A**), ARS quantification (**B**), and calcium content (**C**) of MOVAS treated with the calcifying medium in the presence or absence of Mexo derived from macrophages pretreated with different doses of phosphate (0, 3, 6 mM) (Kruskal–Wallis test, n > 3). Representative images of ARS staining (**D**), ARS quantification (**E**), and calcium content (**F**) of HASMCs treated with the calcifying medium in the presence or absence of Mexo derived from macrophages pretreated with different doses of phosphate (0, 3, 6 mM) (one-way ANOVA, n > 3). (**G**) Calcium content of HASMCs co-cultured with macrophages, in the presence or absence of 3 mM Pi or 20 uM GW4869 (one-way ANOVA, n = 6). (**H**) Schematic diagram of clodronate-liposome-induced macrophage depletion in adenine-induced CKD mouse model. (**I**) Representative images of ARS staining (**I**) and calcium content (**J**) of thoracic aortas of control or CKD mice treated with clodronate (Lipo-Clo) or control liposomes (Lipo-PBS) (Mann–Whitney test, n = 5). * *p* < 0.05, ** *p* < 0.01, *** *p* < 0.001, **** *p* < 0.0001, n.s. not significant. Scale bar, 40 μM.

To investigate the impact of high-phosphate-stimulated macrophage-releasing exosomes on VSMC calcification, macrophage-derived exosomes (Mexo) were isolated following the procedure described in Section 2. The validation of the exosomes was performed using electron microscopy, NTA, and Western blotting (Figure S2). Exosomes from macrophages treated with (Mexo-P) or without (Mexo) extra phosphate were resuspended in PBS and used for further experiments. As shown in Figure 2A–C, the pro-calcification effect of Mexo-P on MOVAS was identified by ARS staining, ARS quantification, and calcium content assay. Similarly, the pro-calcification potential of Mexo-P in HASMC was validated as shown in Figure 2D–F. To explore the role of Mexo-P in macrophage-mediated VSMC calcification in high phosphate, macrophages pretreated with or without exosome suppressor (GW4869) were co-cultured with VSMC in a high-phosphate medium. Figure 2G shows that the GW4869 pretreatment of macrophages significantly decreased high-phosphate-induced VSMC calcium content in this co-culture model. Furthermore, macrophage depletion was induced in the CKD mouse model by using clodronate liposome (Lipo-clo) (Figure 2H). Representative images of ARS staining (Figure 2I) and the results of a calcium content assay (Figure 2J) showed that macrophage depletion significantly mitigated CKD-related calcification in mice aorta. In summary, macrophages and Mexo-P exhibit a pro-calcification potential in high-phosphate-related VSMC calcification in CKD.

### 3.3. Mexo-P Regulates Vascular Calcification in a let-7b-5p-Dependent Manner

**Figure 3:**
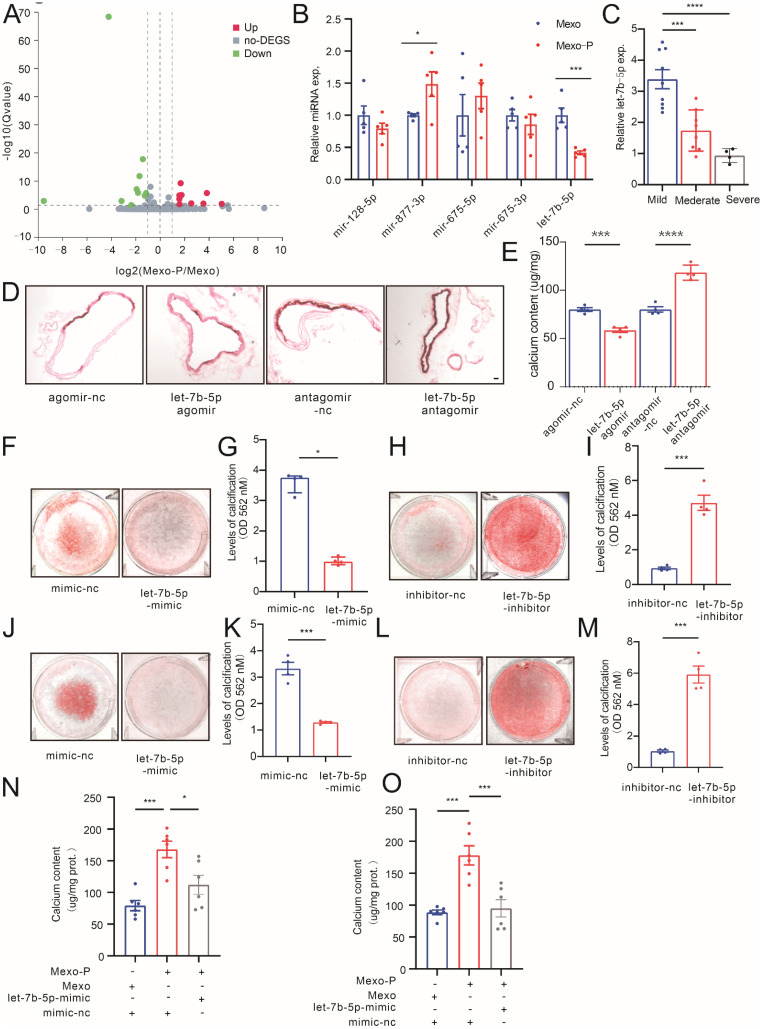
Mexo-P regulates vascular calcification in a let-7b-5p-dependent way. (**A**) Volcano plots show the result of miRNA sequencing between Mexo- and Mexo-P-treated MOVAS. (**B**) qRT-PCR validation of potential target miRNAs between Mexo- and Mexo-P-treated HASMCs (unpaired *t*-test, n = 5). (**C**) Expression levels of let-7b-5p in the artery tissue of mildly, moderately, or severely calcified CKD patients (unpaired *t*-test, n > 3). ARS staining (**D**) and calcium contents ((**E**), one-way ANOVA, n = 4) of isolated mouse thoracic aortas, cultured in a calcifying medium (3 mM Pi) for 7 days, pretreated with let-7b-5p agomir or antagomir for 6 h. Representative images of ARS staining (**F**) and ARS quantification (**G**) of MOVAS treated with let-7b-5p-mimic or control (mimic-nc) and cultured in the calcifying medium for 7 days (Mann–Whitney test, n = 4). Representative images of ARS staining (**H**) and ARS quantification (**I**) of MOVAS treated with let-7b-5p-inhibitor or control (inhibitor-nc) and cultured in the calcifying medium for 7 days (unpaired *t*-test, n = 4). Representative images of ARS staining (**J**) and ARS quantification (**K**) of HASMCs treated with let-7b-5p-mimic or control and cultured in the calcifying medium for 7 days (unpaired *t*-test, n = 4). Representative images of ARS staining (**L**) and ARS quantification (**M**) of MOVAS treated with let-7b-5p-inhibitor or control and cultured in the calcifying medium for 7 days (unpaired *t*-test, n = 4). Calcium content levels of calcifying-medium-cultured VSMCs (MOVAS, (**N**), HASMC, (**O**)), stimulated with Mexo or Mexo-P in the presence or absence of let-7b-5p-mimic (one-way ANOVA, n = 4). * *p* < 0.05, *** *p* < 0.001, **** *p* < 0.0001, n.s. not significant. Scale bar, 40 μM.

To identify the key molecule in Mexo-P regulating VSMC calcification, small RNA sequencing was performed in Mexo- or Mexo-P-stimulated MOVAS. The results showed that 30 miRNAs were significantly differentially expressed between the two groups: 12 were upregulated, and 18 were downregulated (Figure 3A). Of the top 10 downregulated miRNAs, 5 were also expressed in humans. Their expression levels were detected in Mexo- or Mexo-P-stimulated HASMCs. The results showed that two miRNAs, miR-877-3p and let-7b-5p, were differentially regulated, and let-7b-5p, which was chosen as the potential target molecule of Mexo-P, was the only significantly downregulated miRNA (Figure 3B). The expression of let-7b-5p was further identified in CKD patients’ arteries. A remarkably decreased level of let-7b-5p was observed in the artery tissue of CKD-S compared to that of CKD-M (Figure 3C). Next, the pro-calcification potential of let-7b-5p was explored in vivo and ex vivo. In a high-phosphate-induced ex vivo calcification model, the treatment of let-7b-5p-agomir significantly decreased the calcium deposition in mouse arterial rings, while let-7b-5p-antagomir showed the opposite effect (Figure 3D,E). In the high-phosphate-induced VSMC calcification model, let-7b-5p-mimics significantly mitigated the calcification of MOVAS (Figure 3F,G) or HASMCs (Figure 3J,K), while the inhibition of let-7b-5p increased calcium deposition in MOVAS (Figure 3H,I) or HASMCs (Figure 3L,M). Furthermore, the rescue effect of let-7b-5p on VSMCs was explored; the treatment of let-7b-5p-mimics significantly decreased Mexo-P-induced MOVAS or HASMC calcification (Figure 3N,O).

### 3.4. Mexo-P/let-7b-5p Regulate VC through a TGFBR1-Dependent Pathway

**Figure 4:**
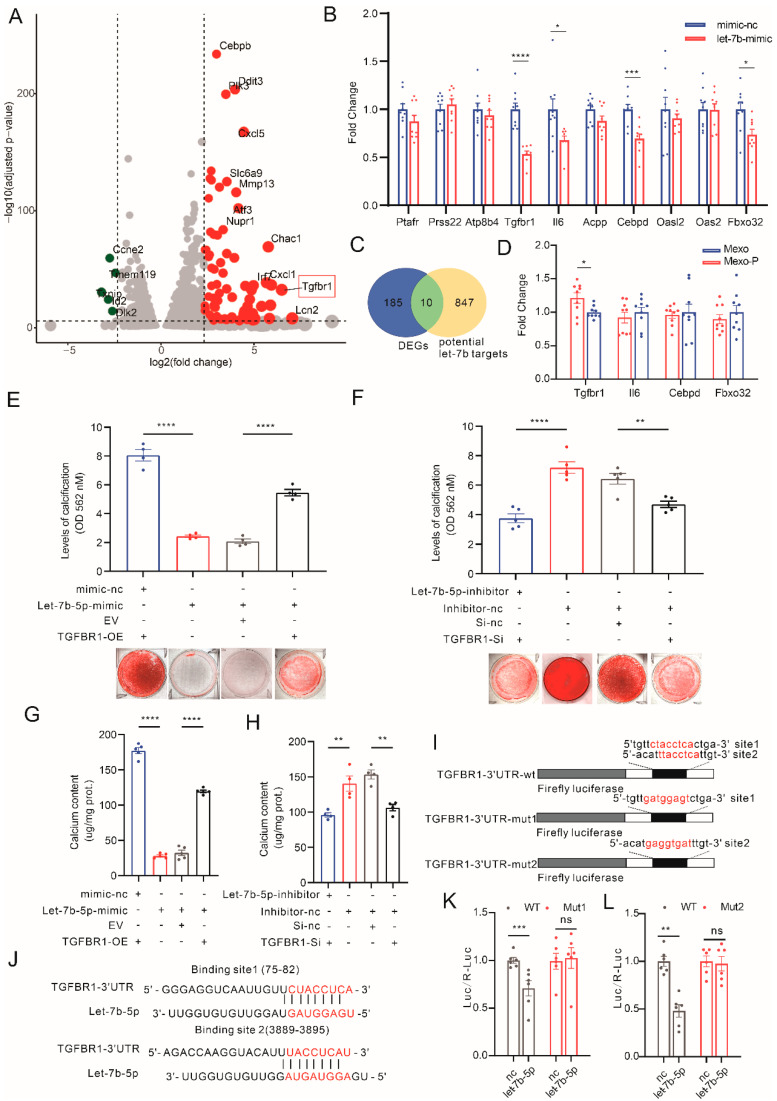
Let-7b-5p mitigates vascular calcification by regulating TGFBR1. (**A**) Volcano plots showing differentially regulated genes (DEGs) in RNA-seq of Mexo- and Mexo-P-treated MOVAS. (**B**) Venn diagram showing the intersection of miRBD-predicted potential let-7b-5p targets and DEGs. (**C**) mRNA levels of 10 potential target genes were detected by qRT-PCR in let-7b-5p-mimic- or mimic-nc-treated HASMCs (unpaired *t*-test, n > 3). (**D**) Transcription levels of four significantly regulated genes in (**C**) were detected in Mexo- or Mexo-P-treated HASMC (unpaired *t*-test, n > 3). (**E**) Representative images of ARS staining (bottom) and ARS quantification (top) of HASMCs, in which TGFBR1 and let-7b-5p levels were manipulated with TGFBR1 overexpression plasmid (TGFBR1-OE) or let-7b-5p-mimic treatment in calcifying medium (one-way ANOVA, n = 4). (**F**) Representative images of ARS staining (bottom) and ARS quantification (top) of HASMCs, in which TGFBR1 and let-7b-5p levels were manipulated with TGFBR1-si or let-7b-5p-mimic treatment in calcifying medium (one-way ANOVA, n = 4). Calcium content of HASMCs with altered TGFBR1 levels with TGFBR1-OE (**G**) or TGFBR1-si (**H**), with or without the treatment of let-7b-5p-mimic or let-7b-5p-inhibitor in calcifying medium (one-way ANOVA, n = 4). (**I**) Tergetscan database predicted binding sequences of let-7b-5p on TGFBR1-3′UTR. (**J**) Wildtype (wt) and two mutations (mut1 and mut2) of TGFBR1-3′UTR were inserted into luciferase vectors. Luciferase activity assay of HASMCs co-transfected with vectors carrying wt or mutated TGFBR1-3′UTR (mut1, (**K**), mut2, (**L**)), renilla vectors, mimic-nc, or let-7b-5p-mimic for 6 h, medium change, and cultured for another 48 h before assay (two-way ANOVA, n > 3). * *p* < 0.05, ** *p* < 0.01, *** *p* < 0.001, **** *p* < 0.0001, n.s. not significant.

To identify targets of let-7b-5p in VSMCs, MOVAS treated with Mexo-P or Mexo was sent for transcriptome analysis. Differentially expressed genes (DEGs) are shown in volcano plots (Figure 4A). Meanwhile, 857 let-7b-5p-regulated genes were predicted using the miRNA database, miRDB (mirdb.org) (Figure 4C). The intersection of these two sets of genes was calculated, and we obtained 10 genes: Ptafr, Prss22, Atp8b4, Tgfbr1, Il6, Acpp, Cebpd, Oasl2, Oas2, and Fbxo32. The transcriptive levels of these 10 genes were then tested in let-7b-5p-mimic-treated HASMCs, 4 of which showed significant downregulation, while others did not (Figure 4B). The expressions of these four genes were then detected in the Mexo-P- or Mexo-treated HASMCs, and only TGFBR1 significantly increased in the Mexo-P-treated group, compared with the Mexo group (Figure 4D). Hence, TGFBR1 was chosen for further analysis. To further confirm that let-7b-5p regulates VC via TGFBR1, TGFBR1 overexpression and knockdown were performed in an in vitro model. TGFBR1-overexpressed HASMCs were treated with control mimics or let-7b-5p-mimics. ARS staining showed that TGFBR1 overexpression rescues let-7b-5p-mimic-mediated VC suppression (Figure 4E,G), while let-7b-5p inhibitors reversed the inhibitory effect of TGFBR1-si on calcification (Figure 4F,H), indicating the regulative role of let-7b-5p/TGFBR1 in VC. The direct regulation of let-7b-5p on TGFBR1 was then studied with a dual-luciferase reporter-gene system. The two binding sites of let-7b-5p on TGFBR1-3′UTR were predicted using the Targetscan online database (Figure 4I). WT and two mutations of these binding sites of TGFBR1-3′UTR were generated and expressed in HASMCs (Figure 4J). Interestingly, both mutations can restore the let-7b-5p-induced downregulation of TGFBR1-3′UTR expression to different degrees. This means that they were both let-7b-5p binding sites on TGFBR1 in HASMCs (Figure 4K,L).

Taken together, a combination of RNA-seq and qRT-PCR identified TGFBR1 as the downstream of Mexo-P/let-7b-5p. Rescue experiments in VSMCs calcification validated the role of TGFBR1 in let-7b-5p-regulating calcification. The binding sites of let-7b-5p on TGFBR1 were identified by a dual-luciferase reporter assay.

### 3.5. TGFBR1 Expression Upregulated in Calcified Media in CKD

**Figure 5:**
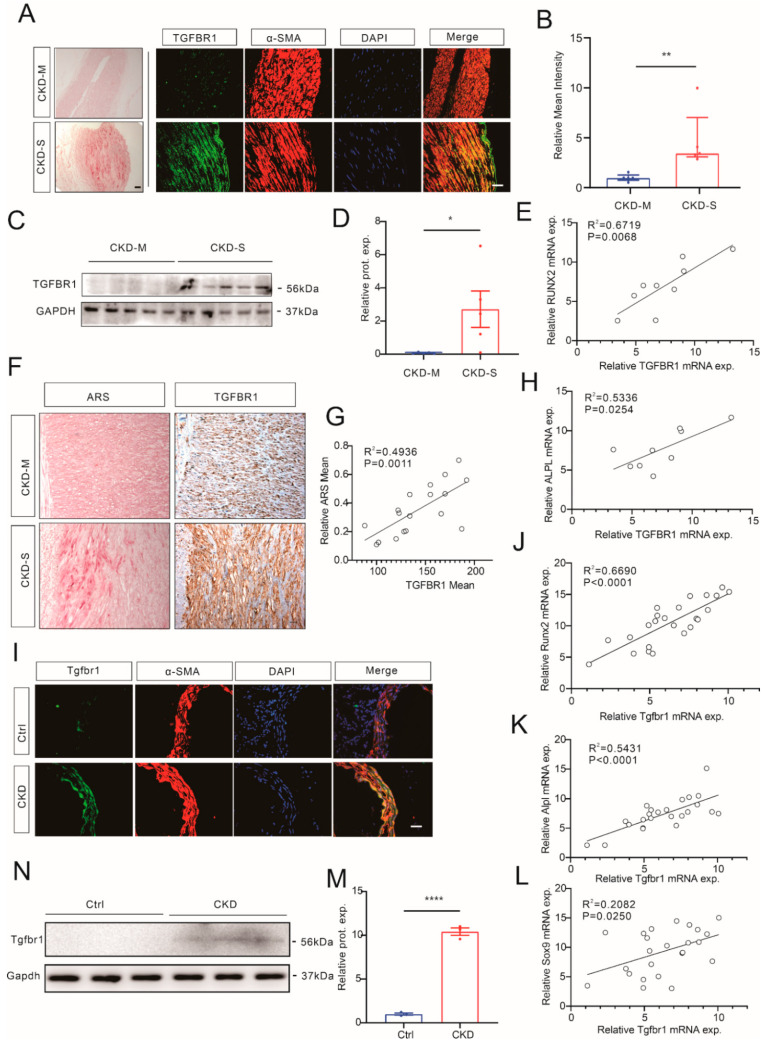
TGFBR1 expression level increased in calcified media of CKD. Representative IF staining (**A**) and semi-quantification (**B**) of TGFBR/α-SMA in arteries from CKD-M and CKD-S groups (bar 40 μM, Mann–Whitney test, n = 5). Representative images (**C**) and semi-quantification (**D**) of Western blot showing TGFBR1 levels in arteries of CKD patients (unpaired *t*-test, n = 5). Correlation of mRNA levels of TGFBR1 and RUNX2 (**E**) or ALPL (**H**) in arterial tissue of CKD patients. (**F**) Representative IHC staining of TGFBR1 and ARS staining of arteries from CKD patients. (**G**) Semi-quantification showing the correlation of intensity mean of TGFBR1 IHC staining and mean area of positive ARS staining in arteries from CKD patients. (**I**) Representative IF staining of Tgfbr1 (green) and α-SMA (red) of aortas from CKD mouse model and control (n = 19). Correlation of mRNA levels of Tgfbr1 and Runx2 (**J**) or Alpl (**K**), as well as Sox9 (**L**) in aortas from CKD mouse model. (**N**) Representative images (**N**) and semi-quantification (**M**) of Western blot showing Tgfbr1 levels in aortas from CKD mouse model (unpaired *t*-test, n = 3). * *p* < 0.05, ** *p* < 0.01, **** *p* < 0.0001, n.s. not significant. Scale bar, 40 μM.

To investigate the expression level of TGFBR1 in CKD arteries, IF, WB, and qRT-PCR of TGFBR1 were performed with arteries from CKD patients. The results showed a significantly increased co-localization of TGFBR1 and a-SMA (Figure 5A,B). The WB results showed that the TGFBR1 protein level was significantly increased in the severely calcified group (Figure 5C,D), while the mRNA level of TGFBR1 was correlated with the transcription levels of RUNX2 and ALPL (Figure 5E,H). To further verify the relationship between TGFBR1 and vascular calcification, IHC of TGFBR1 was performed on arteries from CKD patients. The semi-quantification of the mean of the TGFBR1 positively stained area was found to be positively correlated with the ARS mean (Figure 5F,G). Subsequently, the TGFBR1 expression levels were explored in mice arteries from a mouse CKD model. IF of the mice aorta tissue showed that Tgfbr1 was upregulated in the CKD group (Figure 5I), and WB also showed similar results (Figure 5N,M). The mRNA levels of TGFBR1 were also found to be correlated with calcification-related genes, Runx2, Alpl, and Sox9 (Figure 5J–L).

In short, we found that TGFBR1 was upregulated in the arteries of calcified CKD patients and mice.

### 3.6. Manipulating TGFBR1 Level Regulates Vascular Calcification in CKD

**Figure 6:**
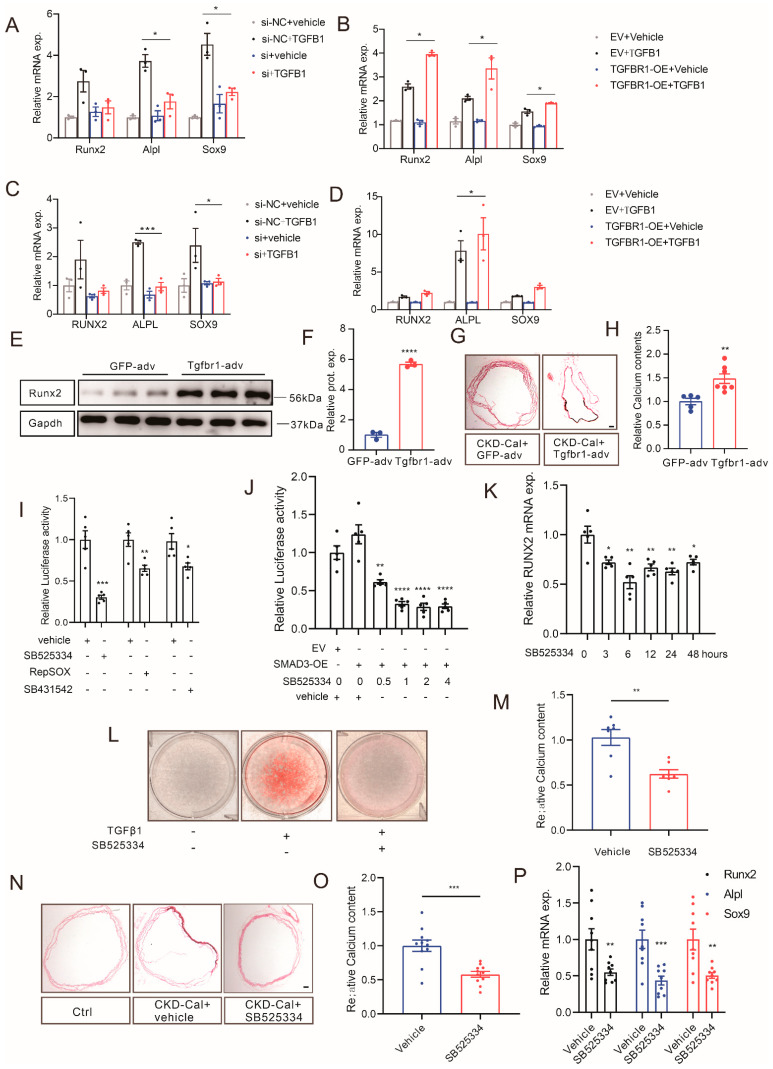
Manipulating TGFBR1 levels regulates vascular calcification in CKD. Transcription levels of RUNX2, ALPL, and SOX9 in MOVAS (**A**) and HASMCs (**C**) stimulated with 10 ng/mL TGFβ1 for 24 h after being transfected with TGFBR1 siRNA for 6 h (one-way ANOVA, n > 3). mRNA levels of RUNX2, ALPL, and SOX9 in MOVAS (**B**) and HASMCs (**D**) stimulated with 10 ng/mL TGFβ1 for 24 h after being transfected with TGFBR1 overexpression plasmid (TGFBR1-OE) for 48 h (one-way ANOVA, n > 3). Representative WB images (**E**) and semi-quantification (**F**) showing RUNX2 levels in aortas from CKD mice injected with either Tgfbr1-adv or control (GFP-adv, unpaired *t*-test, n = 3). Representative images of ARS staining (**G**) and calcium content (**H**) of aortas from CKD mice injected with either Tgfbr1-adv or control (unpaired *t*-test, n > 3). (**I**) HASMCs were treated with SB525334 (1 μM), RepSOX (25 μM), and SB431542 (1 μM) for 6 h, after being transfected with SMAD3-luciferase plasmids for 24 h, and luciferase activity was detected (unpaired *t*-test, n = 3). (**J**) SMAD3-luciferase plasmid-transfected HASMCs were stimulated with 10 ng/mL TGFβ1 and different doses of SB525334 for 6 h, and luciferase activity was detected (one-way ANOVA, n > 3). (**K**) HASMCs were stimulated with 10 ng/mL TGFβ1 plus 1 μM SB525334 for 0, 3, 6, 12, 24, or 48 h, and RUNX2 mRNA levels were detected by qRT-PCR (one-way ANOVA, n = 5). In vitro calcification model showing the SB525334 (1 μM) inhibition of 10 ng/mL TGFβ1-induced HASMC calcification ((**L**), ARS staining, (**M**), calcium content, one-way ANOVA, n = 6). Mice were orally administrated with 30 mg/kg SB525334 (n = 5) or vehicle (n = 5) daily; simultaneously with CKD modeling, aortas were collected for ARS staining (**N**), calcium content assay ((**O**), unpaired *t*-test, n > 3), and Runx2, Alpl, and Sox9 mRNA levels detection ((**P**), unpaired *t*-test, n > 3). * *p* < 0.05, ** *p* < 0.01, *** *p* < 0.001, **** *p* < 0.0001, n.s. not significant. Scale bar, 40 μM.

To explore the role of TGFBR1 in CKD-regulated VC, TGFBR1 knockdown and overexpression were generated in vivo and in vitro, respectively. The expression level of TGFBR1 was knocked down by using siRNA in VSMCs at around 30% (Figure S3F, MOVAS on the left side and HASMCs on the right side). The suppression of this TGFBR1 transcription level mitigated TGFβ1-induced RUNX2, ALPL, and SOX9 mRNA level upregulation in MOVAS and HASMCs (Figure 6A,C). Next, the TGFBR1 overexpression construct (TGFBR1-OE) was generated, which upregulated the expression level of TGFBR1 mRNA by around 2.5 folds in MOVAS and by over 6 folds in HASMCs (Figure S3G, MOVAS on the left side and HASMCs on the right side). Compared to the empty vector (EV), the transfection of TGFBR1-OE plasmids significantly upregulated the TGFβ1-increased mRNA levels of RUNX2, ALPL, and SOX9 in VSMCs (Figure 6B,D).

In the CKD mouse model, Tgfbr1 was overexpressed via injecting adenovirus (Tgfbr1-adv). The results of IF and WB showed that Tgfbr1 was strongly upregulated in mouse aortas 15 days after the injection of Tgfbr1-adv, compared to 5 days (Figure S3C). In comparison to the control GFP-adv group, CKD mice in the Tgfbr1-adv group exhibited elevated calcification in the aortas (Figure 6G,H). WB of the aortas also indicated an increased protein expression of RUNX2 (Figure 6E,F).

Next, TGFBR1 inhibitors were applied in the calcification models. Three inhibitors of TGFBR1, SB525334, RepSOX, and SB431542 were added in SMAD3-luciferase plasmid-transfected HASMCs, individually. All of the three inhibitors reversed TGFβ1-increased SMAD3 expression, and SB525334 had the strongest effect, being able to reduce the luminescence intensity to 40% (Figure 6I). Time and dose gradients of SB525334 were performed to explore the best treatment duration and concentration (Figure 6J). The results showed that SB525334 had the best inhibitory effect in 1 μM and 6 h (Figure 6J,K). Next, SB525334 treatment was found to be able to downregulate the calcium content and ARS intensity in a VSMC calcification model (Figure 6L,M). Furthermore, the calcification inhibition effect of SB525334 was validated in a CKD mouse model. Mice were orally given 10 mg/kg/day SB525334 or an equal volume of vehicle and fed with adenine and a high-phosphate diet to induce CKD and VC. In the TGFBR1 inhibition group, mice were found to have alleviated calcification in the aorta (Figure 6N–P).

In summary, we found that the upregulation of TGFBR1 promoted VC, while the knockdown or inhibition of TGFBR1 ameliorated VC.

### 3.7. TGFBR1 Promotes Vascular Calcification through SMAD3/RUNX2 Pathway

**Figure 7:**
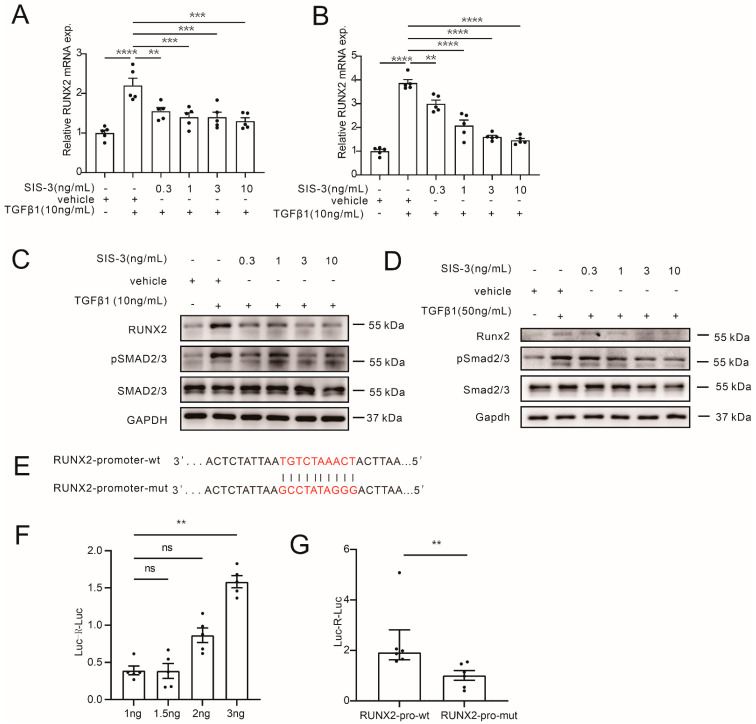
TGFBR1 promotes vascular calcification through the SMAD3/RUNX2 pathway. qRT-PCR results showing that SIS-3 reversed TGFβ1-induced upregulation of RUNX2 levels in HASMCs (**A**) and MOVAS ((**B**), one-way ANOVA, n = 5). Western blot images showing that different doses of SMAD3 inhibitor SIS-3 alleviated TGFβ1-induced RUNX2 level increase and SMAD2/3 phosphorylation in HAMSCs (**C**) and MOVAS (**D**). (**E**) The JASPAR database predicted the binding site of SMAD3 on RUNX2 promoter, and the mutation was generated. (**F**) HASMCs were transfected with *Renilla* plasmid, wildtype RUNX2-promoter luciferase plasmid, and different doses of SMAD3-OE plasmid for 24 h, and luciferase activity was detected (one-way ANOVA, n = 5). (**G**) HASMC was transfected with *Renilla* plasmid, SMAD3-OE plasmid (3 ng per well of 12-well plate), and wildtype (wt) or mutated (mut) RUNX2-promoter luciferase plasmid, and luciferase activity was detected (Mann–Whitney test, n = 6). ** *p* < 0.01, *** *p* < 0.001, **** *p* < 0.0001, n.s. not significant.

We further explored the downstream mechanism through which TGFBR1 regulates calcification. The classic TGFBR1 downstream signaling, SMAD3, was investigated. The treatment of the inhibitor of SMAD3, SIS-3, ameliorated the TGFβ1-induced upregulation of RUNX2 levels (both protein and mRNA) in VSMCs (Figure 7A,C for HASMCs, Figure 7B,D for MOVAS). A Promoter assay was then performed to explore the binding of SMAD3 on the RUNX2 promoter. The JASPAR database predicted one high-score binding site of SMAD3 and RUNX2 promoter, which was then mutated (Figure 7E). Wildtype and mutated RUNX2 promoter sequences were inserted into luciferase plasmid to generate two plasmids, RUNX2-promoter-wt and RUNX2-promoter-mut. In a dual-luciferase system, different doses of SMAD3 overexpression plasmids (SMAD3-OE) were transfected, and RUNX2-promoter-wt was co-transfected. Relative reporter activity (firefly/renilla) showed that 3ng transfection of SMAD3-OE could induce high reporter activity (Figure 7F). Finally, either RUNX2-promoter-wt or RUNX2-promoter-mut was co-transfected with SMAD3-OE in HASMCs. The results showed that RUNX2-promoter-mut significantly alleviated the increased relative luciferase activity in RUNX2-promoter-wt, which indicated that SMAD3 bound the TGTCTAAACT sequence in RUNX2 promoter (Figure 7G).

In summary, we confirmed in HASMCs that TGFβ1 induced RUNX2 upregulation through a SMAD3-dependent pathway. Our results indicated that TGFBR1 regulates calcification by promoting RUNX2 transcription via SMAD3.

## 4. Discussion

In this study, the pro-calcification (pro-cal) effect of high-phosphate-stimulated macrophage-derived exosomes (Mexo-P) was identified in CKD-associated vascular calcification (VC). The pro-cal effect of macrophages has been widely studied in intimal calcification, for instance, atherosclerotic calcification [7,8], but less in media calcification, which is the most common type of VC in CKD. In our present study, we found that the level of macrophage infiltration significantly increased in the media of CKD patients with severe calcification phenotype (Cal-S). Depletion of macrophages in the CKD mouse model relieved VC in mice, further indicating the pro-cal effect of macrophages. The mechanism underlying macrophages regulating media calcification has been investigated in a few studies [13], which reveal the involvement of inflammatory factors/processes, while less is known about the impact of macrophage-derived exosomes (Mexo).

It has been shown that Mexo plays an important role in the pathophysiology of osteoblasts [17], which share similar mechanisms with VC [15,19]. We further investigated the effect of Mexo in VC. As expected, we revealed that exosomes derived from macrophages treated with high phosphate (Mexo-P) were found to be capable of promoting VC in vivo. A recently published study by Boullier [13], which showed that exosomes derived from LPS-treated macrophages aggravated VC, also supported the pro-calcification effect of macrophage-derived exosomes. Exosomes are small, single-membrane, secreted organelles of ∼30 to ∼200 nm in diameter that can transfer proteins, lipids, and non-coding RNAs to achieve cellular communication in both physiological and pathological processes [9,20]. Evidence from previous studies has proved that exosomes are capable of regulating cellular properties via modulating non-coding RNA, such as miRNA, through “sponging-like” procedures [18]. Here, we focused on VSMCs’ intracellular miRNA regulated by Mexo-P during VC.

Let-7b-5p is a miRNA from the let-7 family [21]. The members of the family have been identified as important participants in various cardiovascular diseases, such as cardiac hypertrophy, myocardial infarction [22], and heart failure [23,24,25]. However, the role of let-7b has never been studied in VC. Taking advantage of the miRNA sequencing of Mexo-P-treated VSMCs, we identified let-7b-5p as downstream of Mexo-P in vascular calcification.

In this study, we found that let-7b-5p levels significantly decreased in the arteries of CKD patients from the severely calcified group. Using in vitro and ex vivo functional studies, the anti-calcification function of let-7b-5p has been identified for the first time in this study. Furthermore, rescue experiments showed that let-7b-5p alleviated the pro-calcification effect of Mexo-P, indicating that let-7b was downstream of Mexo-P-regulating VC. Since miRNA can regulate mRNA expression and translation, which has been widely investigated by numerous studies, to systematically understand the gene regulatory role of let-7b-5p, we then further determined the downstream target gene of let-7b-5p utilizing combined analysis of the results of RNA-seq of Mexo-P-treated VSMCs and the online miRNA database. Here, in the current study, we have identified TGFBR1 as a high-potential target of let-7b-5p.

Let-7b-5p has been reported to negatively regulate TGFBR1 in various systems, such as placental trophoblasts [26] and endothelium [27]. In our work, it has also been observed that let-7b-5p exhibits a negative regulatory impact on TGFBR1 expression in VSMCs. The TGFBR1 gene codes the receptor of TGFβ, which is a cytokine and has been proved to be capable of promoting calcification [28]. Li et al.’s study has also shown that TGFBR1 is involved in CKD-associated VC [29]. In this study, we comprehensively investigated the role of TGFBR1 in CKD-related VC in patients, CKD mouse models, and VSMC cells. We identified the pro-cal effect of TGFBR1 in CKD-related VC by the knockdown and upregulation of TGFBR1 in vivo and in vitro. Taken together, Mexo-P/let-7b-5p regulated VC via a TGFBR1-dependent pathway, but the downstream mechanism still needs to be explored.

Furthermore, we found that TGFBR1 mediated the pro-cal effect via its classic downstream SMAD3 signaling [28]. Our results showed that Mexo-P upregulated TGFBR1 levels via downregulated let-7b-5p levels. As a result, SMAD3 became more highly activated, which then acted as a transcription factor of RUNX2 and further promoted its transcription. Nicolai’s study found that the overexpression of SMURF2 reduced the levels of RUNX2 and TGFBR1 in an osteoarthritis system [30], and our investigation added direct information about the relationship between RUNX2 and TGFBR1. Moreover, there is very little research that describes the regulatory effect of SMAD3 on RUNX2 in VC. Therefore, our study provided detailed evidence that the activation of TGFBR1/SMAD3 signaling leads to RUNX2 upregulation. It has been widely acknowledged that RUNX2 is a key transcription factor associated with osteoblast differentiation [31,32], which plays an important role in VC. Therefore, our study illustrated for the first time that Mexo-P upregulated the expression level of TGFBR1 via suppressing let-7b-5p, which further activates SMAD3/RUNX2 signaling and promotes VC occurrence. Our results provide novel insight into the impact of macrophage exosomes on promoting VC and its associated let-7b-5p/TGFBR1/SMAD3 axis in VSMCs, which could be a potential therapeutical target for VC in CKD.

## Data Availability

The data underlying this article will be shared on reasonable request to the corresponding author.

## Supplementary Materials

The following supporting information can be downloaded at: https://www.mdpi.com/article/10.3390/cells12010161/s1, The supplementary files include the original images of WB, Figures S1–S3, Tables S1 and S2, and processed data of miRNA or RNA sequencing results, as well as a detailed method part. Figure S1: Characteristic of CKD mice model: (A) representative images of PAS staining of kidney tissue in CKD (left) and control (right) mice; (B) blood creatine (right) and urea nitrogen (left) levels in CKD and control mice (unpaired *t*-test, n = 20); (C) blood calcium (right) and phosphate (left) levels in CKD and control mice (unpaired *t*-test, n= 20); (D) blood creatine (right) and urea nitrogen (left) levels in CKD mice treated with lipo-clo or lipo-PBS (unpaired *t*-test, n = 5); (E) blood calcium (right) and phosphate (left) levels in CKD treated with lipo-clo or lipo-PBS (unpaired *t*-test, n = 5); scale bar, 40 μM; **** *p* < 0.0001, n.s. not significant; Figure S2: Validation of exosomes. (A) Cup-shaped morphology of purified Mexo (arrowheads) assessed by TEM. (B) The particle size and particle concentration of Mexos were analyzed by nanoparticle tracking analysis (NTA). (C) Representative images of western blotting showing the exosomal pro-tein markers. (D) qRT-PCR results showing let-7b-5p expression levels in arteries of CKD patients (unpaired *t*-test, n > 3); **** *p* < 0.0001, n.s. not significant; Figure S3: Characteristic of CKD mice with TGFBR1 overexpression or inhibition: blood urea nitrogen (A) and creatine (B) levels in CKD mice treated with Tgfbr1-adv or GFP-adv (unpaired *t*-test, n = 5); representative fluorescence microscopy (C) images of aortas from mice injected with Tgfbr1-adv for 5 days or 15 days (n = 5); (D) blood urea nitrogen (left) and creatine (right) levels in CKD mice treated with Tgfbr1-inhibitor or vehicle (unpaired *t*-test, n = 5); (E) blood calcium (right) and phosphate (left) levels in CKD mice treated with Tgfbr1-inhibitor or vehicle (unpaired *t*-test, n = 5); (F) MOVAS (left) or HASMC (right) cells were transfected with TGFBR1 siRNAs, RNA was extracted after 72 h of culture, and TGFBR1 mRNA levels were detected with qRT-PCR; (G) MOVAS (left) or HASMC (right) cells were transfected with TGFBR1 overexpression plasmids (TGFBR1-OE) or empty vehicle (EV), RNA was extracted after 72 h of culture, and TGFBR1 mRNA levels were detected with qRT-PCR; scale bar, 40 μM; * *p* < 0.05, ** *p* < 0.01, **** *p* < 0.0001, n.s. not significant; Table S1:Baseline Characteristics of the Patients; Table S2: Primers of miRNA and RNA [20,33,34,35,36].

## References

[1] Blacher J., Guerin A.P., Pannier B., Marchais S.J., London G.M. (2001). Arterial calcifications, arterial stiffness, and cardiovascular risk in end-stage renal disease. Hypertension.

[2] London G.M., Guérin A.P., Marchais S.J., Métivier F., Pannier B., Adda H. (2003). Arterial media calcification in end-stage renal disease: Impact on all-cause and cardiovascular mortality. Nephrol. Dial. Transplant..

[3] Nelson A.J., Raggi P., Wolf M., Gold A.M., Chertow G.M., Roe M.T. (2020). Targeting Vascular Calcification in Chronic Kidney Disease. JACC Basic Transl. Sci..

[4] Lehto S., Niskanen L., Suhonen M., Rönnemaa T., Laakso M. (1996). Medial artery calcification. A neglected harbinger of cardiovascular complications in non-insulin-dependent diabetes mellitus. Arterioscler. Thromb. Vasc. Biol..

[5] Durham A.L., Speer M.Y., Scatena M., Giachelli C.M., Shanahan C.M. (2018). Role of smooth muscle cells in vascular calcification: Implications in atherosclerosis and arterial stiffness. Cardiovasc. Res..

[6] Lanzer P., Hannan F.M., Lanzer J.D., Janzen J., Raggi P., Furniss D., Schuchardt M., Thakker R., Fok P.W., Saez-Rodriguez J. (2021). Medial Arterial Calcification: JACC State-of-the-Art Review. J. Am. Coll. Cardiol..

[7] Waring O.J., Skenteris N.T., Biessen E.A.L., Donners M. (2021). Two-faced Janus: The dual role of macrophages in atherosclerotic calcification. Cardiovasc. Res..

[8] Li C., Qu L., Matz A.J., Murphy P.A., Liu Y., Manichaikul A.W., Aguiar D., Rich S.S., Herrington D.M., Vu D. (2022). AtheroSpectrum Reveals Novel Macrophage Foam Cell Gene Signatures Associated With Atherosclerotic Cardiovascular Disease Risk. Circulation.

[9] Pluchino S., Smith J.A. (2019). Explicating Exosomes: Reclassifying the Rising Stars of Intercellular Communication. Cell.

[10] Théry C., Witwer K.W., Aikawa E., Alcaraz M.J., Anderson J.D., Andriantsitohaina R., Antoniou A., Arab T., Archer F., Atkin-Smith G.K. (2018). Minimal information for studies of extracellular vesicles 2018 (MISEV2018): A position statement of the International Society for Extracellular Vesicles and update of the MISEV2014 guidelines. J. Extracell. Vesicles.

[11] Das S., Halushka M.K. (2015). Extracellular vesicle microRNA transfer in cardiovascular disease. Cardiovasc. Pathol..

[12] Nazarenko I., Rupp A.K., Altevogt P. (2013). Exosomes as a potential tool for a specific delivery of functional molecules. Methods Mol. Biol..

[13] Ismail N., Wang Y., Dakhlallah D., Moldovan L., Agarwal K., Batte K., Shah P., Wisler J., Eubank T.D., Tridandapani S. (2013). Macrophage microvesicles induce macrophage differentiation and miR-223 transfer. Blood.

[14] Yaker L., Tebani A., Lesueur C., Dias C., Jung V., Bekri S., Guerrera I.C., Kamel S., Ausseil J., Boullier A. (2022). Extracellular Vesicles From LPS-Treated Macrophages Aggravate Smooth Muscle Cell Calcification by Propagating Inflammation and Oxidative Stress. Front. Cell Dev. Biol..

[15] Chen X., Wan Z., Yang L., Song S., Fu Z., Tang K., Chen L., Song Y. (2022). Exosomes derived from reparative M2-like macrophages prevent bone loss in murine periodontitis models via IL-10 mRNA. J. Nanobiotechnol..

[16] Zhu J., Liu B., Wang Z., Wang D., Ni H., Zhang L., Wang Y. (2019). Exosomes from nicotine-stimulated macrophages accelerate atherosclerosis through miR-21-3p/PTEN-mediated VSMC migration and proliferation. Theranostics.

[17] Wang Z., Zhu H., Shi H., Zhao H., Gao R., Weng X., Liu R., Li X., Zou Y., Hu K. (2019). Exosomes derived from M1 macrophages aggravate neointimal hyperplasia following carotid artery injuries in mice through miR-222/CDKN1B/CDKN1C pathway. Cell Death Dis..

[18] Yan W., Li T., Yin T., Hou Z., Qu K., Wang N., Durkan C., Dong L., Qiu J., Gregersen H. (2020). M2 macrophage-derived exosomes promote the c-KIT phenotype of vascular smooth muscle cells during vascular tissue repair after intravascular stent implantation. Theranostics.

[19] Li R., Li D., Wang H., Chen K., Wang S., Xu J., Ji P. (2022). Exosomes from adipose-derived stem cells regulate M1/M2 macrophage phenotypic polarization to promote bone healing via miR-451a/MIF. Stem Cell Res. Ther..

[20] Xu Z., Chen Y., Ma L., Chen Y., Liu J., Guo Y., Yu T., Zhang L., Zhu L., Shu Y. (2022). Role of exosomal non-coding RNAs from tumor cells and tumor-associated macrophages in the tumor microenvironment. Mol. Ther. J. Am. Soc. Gene Ther..

[21] Bao M.H., Feng X., Zhang Y.W., Lou X.Y., Cheng Y., Zhou H.H. (2013). Let-7 in cardiovascular diseases, heart development and cardiovascular differentiation from stem cells. Int. J. Mol. Sci..

[22] Li J., Li K., Chen X. (2019). Inflammation-regulatory microRNAs: Valuable targets for intracranial atherosclerosis. J. Neurosci. Res..

[23] Luo X., Zhang H., Xiao J., Wang Z. (2010). Regulation of human cardiac ion channel genes by microRNAs: Theoretical perspective and pathophysiological implications. Cell. Physiol. Biochem..

[24] Thum T., Galuppo P., Wolf C., Fiedler J., Kneitz S., van Laake L.W., Doevendans P.A., Mummery C.L., Borlak J., Haverich A. (2007). MicroRNAs in the human heart: A clue to fetal gene reprogramming in heart failure. Circulation.

[25] Topkara V.K., Mann D.L. (2011). Role of microRNAs in cardiac remodeling and heart failure. Cardiovasc. Drugs Ther..

[26] Gao Y., Zhang X., Meng T. (2022). Overexpression of let-7b exerts beneficial effects on the functions of human placental trophoblasts by activating the ERK1/2 signaling pathway. Mol. Reprod. Dev..

[27] Liu X., Li S., Yang Y., Sun Y., Yang Q., Gu N., Li J., Huang T., Liu Y., Dong H. (2021). The lncRNA ANRIL regulates endothelial dysfunction by targeting the let-7b/TGF-βR1 signalling pathway. J. Cell. Physiol..

[28] Vander Ark A., Cao J., Li X. (2018). TGF-β receptors: In and beyond TGF-β signaling. Cell. Signal..

[29] Li C., Zhang S., Chen X., Ji J., Yang W., Gui T., Gai Z., Li Y. (2020). Farnesoid X receptor activation inhibits TGFBR1/TAK1-mediated vascular inflammation and calcification via miR-135a-5p. Commun. Biol..

[30] Schminke B., Kauffmann P., Schubert A., Altherr M., Gelis T., Miosge N. (2021). SMURF1 and SMURF2 in Progenitor Cells from Articular Cartilage and Meniscus during Late-Stage Osteoarthritis. Cartilage.

[31] Hao J., Tang J., Zhang L., Li X., Hao L. (2020). The Crosstalk between Calcium Ions and Aldosterone Contributes to Inflammation, Apoptosis, and Calcification of VSMC via the AIF-1/NF-κB Pathway in Uremia. Oxidative Med. Cell. Longev..

[32] Komori T. (2018). Runx2, an inducer of osteoblast and chondrocyte differentiation. Histochem. Cell Biol..

[33] Zhang Y., Zhang C., Li L., Liang X., Cheng P., Li Q., Chang X., Wang K., Huang S., Li Y. (2021). Lymphangiogenesis in renal fibrosis arises from macrophages via VEGF-C/VEGFR3-dependent autophagy and polarization. Cell Death Dis..

[34] Shi J., Yang Y., Wang Y.N., Li Q., Xing X., Cheng A.Y., Zhan X.N., Li J., Xu G., He F. (2022). Oxidative phosphorylation promotes vascular calcification in chronic kidney disease. Cell Death Dis..

[35] Long X., Yao X., Jiang Q., Yang Y., He X., Tian W., Zhao K., Zhang H. (2020). Astrocyte-derived exosomes enriched with miR-873a-5p inhibit neuroinflammation via microglia phenotype modulation after traumatic brain injury. J. Neuroinflamm..

[36] Jiao Y., Li W., Wang W., Tong X., Xia R., Fan J., Du J., Zhang C., Shi X. (2020). Platelet-derived exosomes promote neutrophil extracellular trap formation during septic shock. Crit. Care.

