# Supplementary material for: High-Phosphate-Stimulated Macrophage-Derived Exosomes Promote Vascular Calcification via let-7b-5p/TGFBR1 Axis in Chronic Kidney Disease
Source: Cells. 2022 Dec 30;12(1):161. doi: 10.3390/cells12010161 (PMC9818696; doi:10.3390/cells12010161)
Supplement: Supplementary file 1 [file cells-12-00161-s001.zip › cells-2091145 detailed method revised.pdf]

Methods

1. Reagents, chemicals, and antibodies

TGFβ1 (HY-P70543, HY-P7117), SB525334 (HY-12043), RepSOX (HY-13012) , SB431542 (HY-10431), SIS-3 (HY-13013) were purchased from MedChemExpress (New Jersey, USA). The trizol reagent was purchased from Takara (Japan), and reverse transcription system kit and SYBR Green PCR master mix kit was obtained from Vazyme (Nanjing, China). Calcium assay kit (C004-2-1) was purchased from Nanjing Jiancheng (Nanjing, China). Antibody of CD68 (MA5-13324), F4/80 (MA1-91124), TGFBR1 (PA5-98192), α-SMA (MA1-06110), RUNX2 (PA5-105643) were obtained from Invitrogen (California, USA). Other antibodies, including SMAD2/3 (A18674), pSMAD2/3 (AP0548), GAPDH (A19056) were obtained from Abclonal (Wuhan, China). Liposome-Clodronate (40337ES08) and Liposome-PBS (40338ES10) were purchased from Yeasen (Shanghai, China).

2. Patient artery samples

A cohort of 21 patients (12 males) with end stage renal diseases undergoing arteriovenous fistula operation was enrolled at Tongji Hospital from Janurary to December 2021. The exclusion criteria were age < 18 years old, diabetes, HIV, and malignant tumors. The artery samples of the 21 patients were acquired during the operation. Moreover, blood samples were collected from patients with advanced CKD (Stage3-5). The CAC score calculation was performed based on chest computed tomography following the protocol described in Tang Z. et al.'s work (1). CKD Patients were divided into three groups based on CAC scores, mildly calcified (CAC 0-99 scores), moderately calcified (CAC 100-399 scores), severely calcified (CAC > 400 scores). The Baseline demographics and clinical characteristics of CKD patients were shown in Table S1.

For use of human plasma and artery tissue specimens, patient consent was obtained as approved by Ethics Committee of Tongji Medical College, Huazhong University of Science and Technology (TJ-IRB20211119), The investigations conform to the principles outlined in the Declaration of Helsinki.

3. Cell culture and treatments

Mouse vascular smooth muscle cells (MOVAS) cell line (CRL-2797TM), and Human aortic smooth muscle cells (HASMC) cell strain (PCS-100-012TM) were from American Type Culture Collection (ATCC, Rockville, MD, USA), and cultured with Dulbecco's Modified Eagle's Medium (DMEM) supplied with 10% fetal bovine serum (FBS) and 1% Penicillin-Streptomycin Solution (P/S, 10kU/mL/10mg/mL). Human aortic. Human monocytic cell line THP-1 (TIB-202™) was also from ATCC and kindly offered by Dr. X Liu (Neurosurgery department, Tongji hospital), the culture medium was RPMI 1640 medium (10% FBS and 1% P/S). For calcification experiments, cells were treated with inorganic phosphate (3 mmol/L referred to as a calcification medium) in the form of Na_2HPO_4/NaH_2PO_4 (pH 7.4) for 24 hours (Realtime-qPCR) or 3 days (calcium deposition quantification, and western blot), or 7days (alizarin red staining). Fresh media with agents were replaced every 2-3 days. For human plasma study, HASMCs were treated with 2.5% plasma. Primary bone-marrow-derived macrophages (BMDMs) from C57BL/6 mice were prepared and cultured followed the protocol described in previous work (2).

4. Aortic ring culture

Aortic tissues were removed from male C57BL/6 mice in a sterile manner. After the adventitia and adipose tissues were removed, the aortas were cut into 2-3 mm rings and cultured in DMEM containing 15% fetal bovine serum (FBS) at 37°C in 5% CO_2. To induce calcification, aortic rings were treated with high-phosphate (3mmol/L) for 14 days. The medium was replaced every 3 days.

5. Animal models.

Adenine induced mice CKD models were both used in this study, and high phosphorus diet were adapted to induce calcification. 8-week-old male C57BL/6 mice were treated with adenine (200mg/kg) via gavage administration daily, and fed with 1.8% phosphorus diet for 5 weeks. The animals were anesthetized i.p. with 90mg/kg ketamine and 10mg/kg xylazine, anaesthesia depth was checked by toe pinch. Blood was drawn via the heart and killing of animals was achieved by cervical dislocation under deep anaesthesia. Animal experiments were approved by Animal Care and Ethics Guidelines (HZAUMO-2021-0152).and performed in accordance with the guidelines from Directive 2010/63/EU of the European Parliament on the protection of animals used for scientific purposes or the NIH Guide for the Care and Use of Laboratory Animals.

6. Calcification analysis

The quantification of VSMCs calcification was performed by incubation of the VSMCs overnight at 4°C in 0.6M HCL. Calcium content was measured with Calcium assay kit and the results were normalized to protein concentration. Alizarin red staining was performed in aortas and VSMCs. Paraformaldehyde-fixed thoracic aortic tissues were stained with 2% alizarin red solution after deparaffinization and rehydration. For VSMCs experiments, cells were fixed and then stained with 2% alizarin red solution.

7. MiRNA intervention

Let-7b mimic, inhibitor, agomir, antagomir and negative controls were synthesized by Augct (Beijing,China). Transfection of let-7b mimics and inhibitors, as well as agomir and antagomir were performed using Lipofectamine3000™ reagent（Invitrogen, CA, USA）following manufacture's instruction.

8. qRT-PCR analysis of mRNA and miRNA.

Total RNA was extracted from the VSMCs or tissues using TRIzol (Invitrogen) and then reverse transcripted into complementary DNA by the reverse transcription system kit (Vazyme). Each expression level was detected by SYBR master-mix (Vazyme). Gene products were then amplified by qPCR on an ABI-Prism 7500 Real-Time PCR System (Applied Biosystems, Carlsbad, CA, USA). The relative level of let-7b was normalized to the expression of control U6 snRNA. Other mRNAs were normalized to the internal standard GAPDH in mice or β-actin in human. The designed primers are listed in Table S2. Data were analyzed using the $2^{-\Delta\Delta}$Ct method.

9. Knockdown and overexpression of TGFBR1

Knockdown of TGFBR1 were performed via transfection of small interfering RNAs (siRNAs) which were synthesized by Augct (Beijing, China). Overexpression plasmids were generated and obtained from Augct. In vivo overexpression of TGFBR1 was carried out with tail vein injection of TGFBR1-OE adenoviruses generated by Obio (Shanghai, China).

10. Immunohistochemistry

Collected vascular tissues were fixed in 4% paraformaldehyde and embedded in paraffin as previously described Shi J et al. (3). After deparaffinization and rehydration, antigen retrieval was performed by citrate buffer (PH 6.0).

For immunohistochemistry, aortic sections were blocked with H_2O_2 for 15 min followed by 10% of goat serum for 1 hour at room temperature, and then incubated overnight at 4°C with primary antibodies (anti-TGFBR1, Invitrogen, 1:200). Vascular sections were washed with PBST and then stained with horseradish peroxidase (HRP)-labeled secondary antibodies. Next, developing was achieved in all samples at the same time with 3,3-diaminobenzidine (DAB), and sections were counterstained with hematoxylin. The light microscope was used to visualize the stained sections. With respect to quantification, random fields were taken for further analysis by ImageJ 1.8.0 (NIH,USA).

For Immunofluorescent, aortic tissues were incubated with primery antibodies (anti-TGFBR1, 1:200, anti-a-SMA, 1:50, anti-CD68, 1:200, anti-F4/80, 1:200) overnight at 4 °C and then incubated with conjugated secondary antibody for 1 h at room temperature in the dark. After several washes with PBS, slides were incubated with DAPI for 3 min and then mounted in glycerol. Slices were imaged with an Olympus Fluoview laser scanning confocal microscope (Olympus, Tokyo, Japan). ImageJ 1.8.0 (NIH) software was used to quantify the intensity of slices.

11. Exosome isolation and validation

The isolation of macrophage-derived exosomes was performed by differential ultracentrifugation according to previous protocols (4). Briefly, macrophages (48hour-PMA treated THP-1 for human experiments, BMDMs for mouse experiments), were cultured in medium supplied with (for Mexo-P) or without 3.0 mM phosphate (for Mexo) for 24 hours. Whereafter, the medium was changed to exosome free culture medium and cultured for another 12 hours. The medium was then collected and centrifuged and 300×g for 5 min at 4 °C, 1000×g for 20 min, and then at 10,000×g for 30 min, sequentially. Then, the supernatants were centrifuged at 100,000g for 70 min. Next, the exosome pellet was washed with PBS and centrifuged at 4 °C and 100,000×g for 70 min. The collected exosomes were resuspended in PBS and then preserved at −80 °C. The sizes and concentrations of the final exosomes were examined by nanoparticle tracking analysis (NTA) using a NanoSight (NS300) measurement. The exosome morphology was measured, as previously described (4), by transmission electron microscopy (TEM, Hitachi H7700, Japan).

12. Western blot assays.

Total protein from artery tissues and VSMCs was extracted in RIPA buffer supplemented with protease and phosphatase inhibitors. Proteins were separated by 10% sodium dodecyl sulfate polyacrylamide gel electrophoresis (SDS-PAGE) and transferred to 0.45μm polyvinylidene fluoride (PVDF) membranes. The membranes were blocked with 5% milk and then incubated with the primary antibodies targeting following targets overnight at 4℃: TGFBR1 (Invitrogen, 1:1000), RUNX2 (Invitrogen, 1:1000), pSMAD2/3 (Abconal, 1:1000), SMAD2/3 (Abconal, 1:1000), CD9 (Abcam, 1:1000), CD 63 (Abcam, 1:1000) and GAPDH (Abconal, 1:4000). Then, the membranes were washed by TBST and incubated with corresponding HRP-conjugated second antibodies (Abconal, 1:5000) for 1.5h at room temperature, and the proteins were visualized by a Genegnome XRQ (Syngene,UK) using enhanced chemiluminescence (ECL) method. The relative protein levels were calculated by normalizing to the loading control by using Image J.

13. Transcriptome sequencing analysis

RNA samples were extracted from MOVAS, which were treated with exosomes isolated from supernatant of macrophages simulated either with serum form CKD mice or from control mice. Sequencing was performed on the DNBSEQ platform. Then, the sequencing reads were processed with the determination of quality using the SOAPnuke tool. The clean reads were mapped to the reference genome using HISAT2 (v2.0.4). The heatmap was drawn by pheatmap (v1.0.8) according to the gene expression in different samples. Essentially, differential expression analysis was performed. To take insight to the change of phenotype, gene ontology (GO), Kyoto Encyclopedia of Genes and Genomes (KEGG) enrichment analysis, and gene set enrichment analysis (GSEA) of annotated different expressed gene were performed. The significant levels of terms and pathways were corrected by Q value with a rigorous threshold (Q value ≤ 0.05) by Bonferroni.

14. miRNA sequencing and date analysis

MOVAS treated with Mexo or Mexo-P for 24 hours, RNA was isolated and send for miRNA sequencing followed the method described in Shi X et al.'s work (5) .

15. Plasmid constructs and reporter assay.

The promoter of the coding sequence of the RUNX2 gene, or the coding sequence of SMAD3 mRNA was subcloned into the GV208 luciferase reporter vector (GeneChem, Shanghai, China). The 3'UTR of TGFBR1 mRNA was inserted into the pMIR-REPORT Luciferase miRNA expression reporter vector (Obio, Shanghai, China). SMAD3 overexpression plasmid (GV712) was purchased from CeneChem. Luciferase reporter constructs were co-transfected into VSMCs with an internal control plasmid, GV045(Renilla luciferase reporter plasmid, Promega), followed by the indicated stimulation. Then, cells were harvested and lysed, and the luciferase activity was determined with the Dual Luciferase Reporter Assay Kit (Promega, USA) according to the manufacturer's instructions.

17. Measurement of covariates from human plasma samples

Enzyme-linked immunosorbent assays (ELISA) were performed according to the manufacturers' instructions to measure FGF-23 (Abcam, USA), soluble Klotho (R&D, USA), Osteoprotegerin (Merck, Germany), Fetuin-A (Abcam, USA) from plasma of patients. Other parameters were from the lab values collected from Tongji Hospital.

16. Statistical analysis

Statistical analysis was conducted using GraphPad Prism version 8.0 software (Graph software, USA). If normally distributed, values for continuous variables with normal distribution are provided as mean (standard deviation). Otherwise, they are provided as median (interquartile range). One-way analysis of variance, unpaired t-test, Mann-Whitney U test, or Kruskal-Wallis test was used for continuous data as appropriate. Chi-square tests or Fisher's exact tests were used for categorical variables. The number of samples for each data (n) is mentioned in figure legends. The statistical significance is expressed as follows: *P<0.05; **P<0.01; ***P<0.001; ****P<0.0001; and n.s., not significant.

1. Zhang, Y.; Zhang, C.; Li, L.; Liang, X.; Cheng, P.; Li, Q.; Chang, X.; Wang, K.; Huang, S.; Li, Y.; et al. Lymphangiogenesis in renal fibrosis arises from macrophages via VEGF-C/VEGFR3-dependent autophagy and polarization. Cell Death Dis. 2021, 12, 109.
2. Shi, J.; Yang, Y.; Wang, Y.N.; Li, Q.; Xing, X.; Cheng, A.Y.; Zhan, X.N.; Li, J.; Xu, G.; He, F. Oxidative phosphorylation promotes vascular calcification in chronic kidney disease. Cell Death Dis. 2022, 13, 229.
3. Long, X.; Yao, X.; Jiang, Q.; Yang, Y.; He, X.; Tian, W.; Zhao, K.; Zhang, H. Astrocyte-derived exosomes enriched with miR-873a-5p inhibit neuroinflammation via microglia phenotype modulation after traumatic brain injury. J. Neuroinflamm. 2020, 17, 89.
4. Jiao, Y.; Li, W.; Wang, W.; Tong, X.; Xia, R.; Fan, J.; Du, J.; Zhang, C.; Shi, X. Platelet-derived exosomes promote neutrophil extracellular trap formation during septic shock. Crit. Care 2020, 24, 380.
