# Supplementary material for: High-Phosphate-Stimulated Macrophage-Derived Exosomes Promote Vascular Calcification via let-7b-5p/TGFBR1 Axis in Chronic Kidney Disease
Source: Cells. 2022 Dec 30;12(1):161. doi: 10.3390/cells12010161 (PMC9818696; doi:10.3390/cells12010161)
Supplement: Supplementary file 1 [file cells-12-00161-s001.zip › cells-2091145 Table S1 revised.pdf]

Table S1. Baseline Characteristics of the Patients.

	Mildly calcified	Moderately calcified	Severely calcified	P
N(Male)	9(5)	8(5)	4(2)	n.s.
Age (years)	46.9 (4)	59.3 (3.3)	63.3 (4.4)	<0.0001
Smoking, ever	5 (55.6%)	4 (50%)	2 (50%)	n.s.
Diabetes, n (%)	2(22.2%)	3(37.5%)	1(25%)	0.0284
Hypertension, n (%)	7 (77.8%)	5 (62.5%)	4 (100%)	<0.0001
Systolic pressure (mmHg)	142.9 (5.6)	139.1 (3.5)	140.8 (18.7)	n.s.
Diastolic pressure (mmHg)	90.1 (4.5)	80.1(3.7)	84.5 (7.7)	0.0022
**Laboratory findings**				
Platelet (10^9/L)	201.7(20.5)	218.3 (23.4)	187.5 (37.3)	n.s.
Blood urea nitrogen (mmol/L)	25.3 (5.6)	19.9 (3.4)	22.7 (3.2)	n.s.
Serum creatinine (μmol/L)	698.8 (164.7)	691 (147.5)	633.5 (277.4)	n.s.
**Cholesterol**				
TC (mmol/L)	4.1 (0.8)	3.9 (0.8)	4.2 (0.9)	n.s.
HDL-C (mmol/L)	0.8 (0.1)	1.6 (0.3)	0.9 (0.1)	<0.0001
LDL-C (mmol/L)	2.2 (0.3)	2.6 (0.6)	2.3 (0.4)	n.s.
Triglycerides (mmol/L)	1.3 (0.2)	2 (0.1)	1.7 (0.3)	<0.0001
**Mineral Metabolites**				
Calcium (mmol/L)	2.1 (0)	2.6 (0.3)	2.1 (0.1)	<0.0001
Adjusted calcium (mmol/L)	2.2 (0)	2.3 (0.1)	2.2 (0)	0.0081
Phosphorus (mmol/L)	4.8 (0.3)	3.6 (0.5)	4.8 (0.3)	<0.0001
Parathyroid hormone (ng/L)	270.3 (69.4)	180.1 (64.8)	425.1 (276.3)	0.0204
FGF-23 (pg/mL)	211.1(133.0-315.6)	215.4 (150.8-291.0)	232.5 (130.4-299.6)	<0.0001
1.25$(OH)_2D$ (pg/mL)	26.5 (10.6)	24.6 (12.3)	25.9 (10.7)	n.s.
Klotho (pg/mL)	360.0 (275.1-491.6)	366.1 (257.8-510.9)	375.1 (280.7-477.9)	n.s.
**Vascular calcification inhibitors**				
Albumin (g/dL)	3.5 (0.6)	3.5 (0.5)	3.6 (0.4)	n.s.
Osteoprotegerin (pmol/L)	10.6 (5.2)	11.0 (4.8)	10.2 (5.4)	n.s.
Fetuin-A (mg/L)	500.6 (166.9)	488.6 (150.1)	476.6 (156.6)	n.s.
**Duration of Hemodialysis**				
0 month	6 (66.7%)	5(67.5%)	3(75.0%)	n.s.
0-6 months	2 (22.2%)	2(25.0%)	0(0.0%)	<0.0001
6 months-1year	1 (11.1%)	1(12.5%)	1(25.0%)	0.0334
**CVD**				
CHD	3 (33.3%)	3 (37.5%)	3 (75.0%)	<0.0001
Stroke	0 (0%)	1 (12.5%)	1 (25.0%)	<0.0001
CHF	3 (33.3%)	2 (25.0%)	1 (25.0%)	0.3444
CAC score	35.1 (13)	272.6 (23.3)	780 (91.4)	<0.0001

Abbreviations: TC, total cholesterol; HDL-C, High-density lipoprotein-cholesterol LDL-C, low-density lipoprotein-cholesterol; FGF23, fibroblast growth factor-23; CVD, cardiovascular disease; CHD, coronary heart disease; CHF, congestive heart failure. Note: If normally distributed, values for continuous variables with normal distribution are provided as mean (standard deviation). Otherwise, they are provided as median (interquartile range). Categorical variables are presented as number (percentage). P values for differences across groups were obtained by one-way analysis of variance, Kruskal-Wallis test, or $\chi2$ test as appropriate. .
