# Supplementary material for: High-Phosphate-Stimulated Macrophage-Derived Exosomes Promote Vascular Calcification via let-7b-5p/TGFBR1 Axis in Chronic Kidney Disease
Source: Cells. 2022 Dec 30;12(1):161. doi: 10.3390/cells12010161 (PMC9818696; doi:10.3390/cells12010161)
Supplement: Supplementary file 1 [file cells-12-00161-s001.zip › cells-2091445 sup figure .pdf]

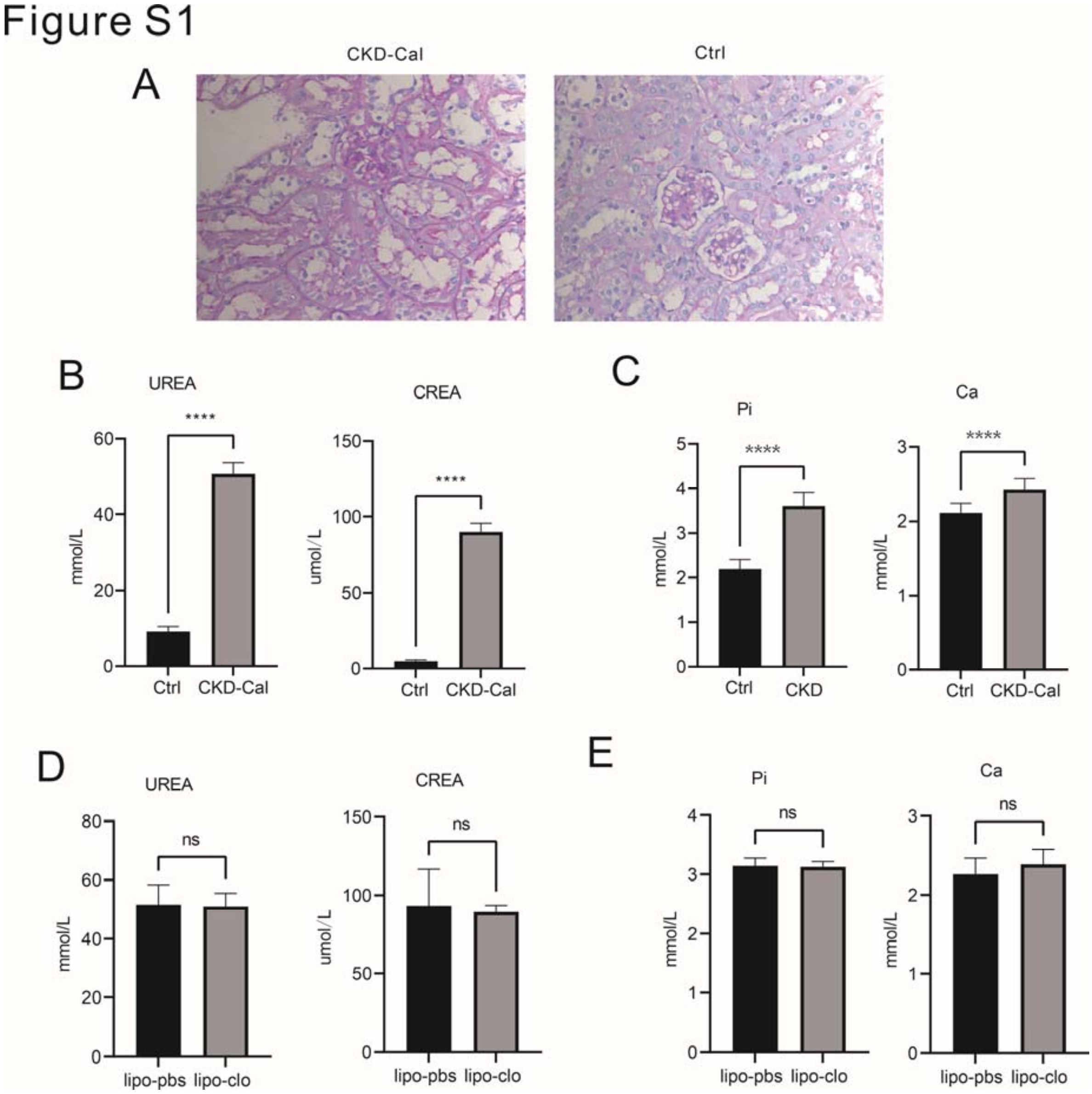

Figure S1. Characteristic of CKD mice model: (A) representative images of PAS staining of kidney tissue in CKD (left) and control (right) mice; (B) blood creatine (right) and urea nitrogen (left) levels in CKD and control mice (unpaired *t*-test, n = 20); (C) blood calcium (right) and phosphate (left) levels in CKD and control mice (unpaired *t*-test, n= 20); (D) blood creatine (right) and urea nitrogen (left) levels in CKD mice treated with lipo-clo or lipo-PBS (unpaired *t*-test, n = 5); (E) blood calcium (right) and phosphate (left) levels in CKD treated with lipo-clo or lipo-PBS (unpaired *t*-test, n = 5); scale bar, 40 μM; **** $p < 0.0001$, n.s. not significant.

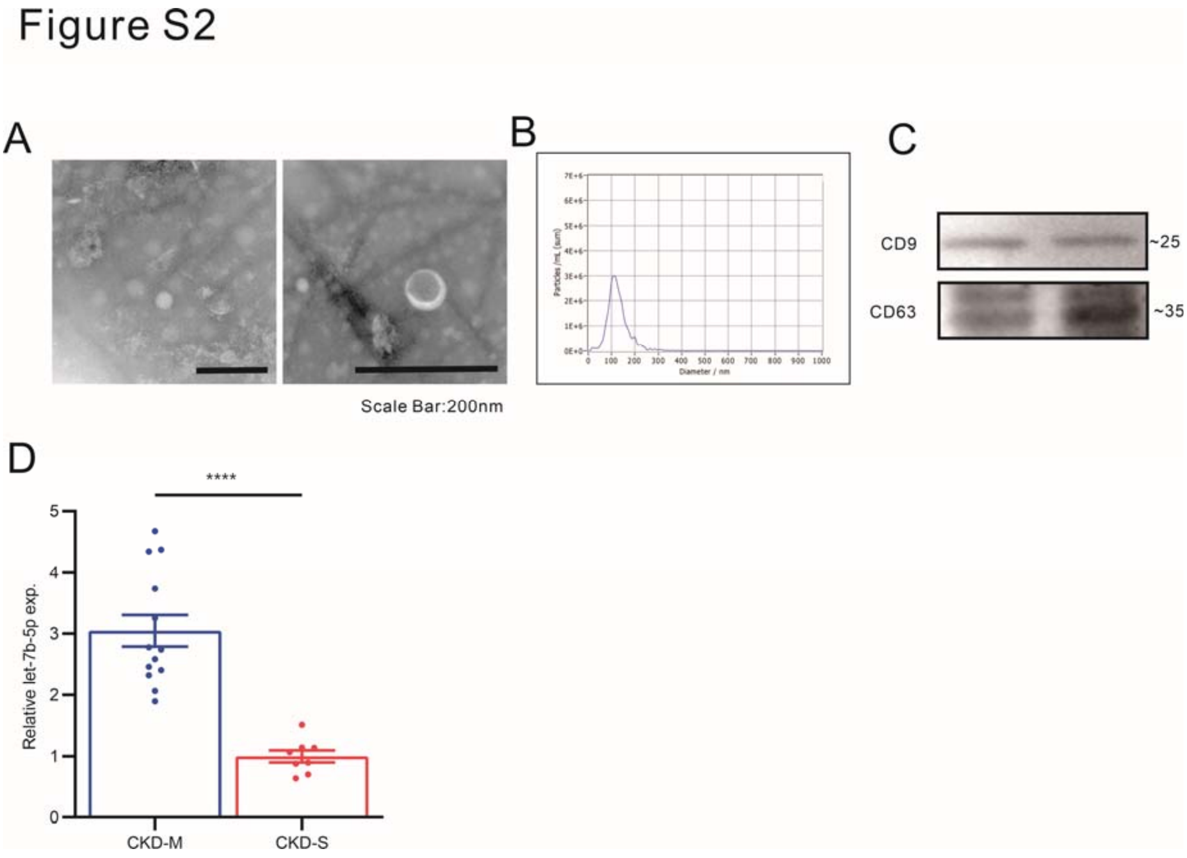

Figure S2. Validation of exosomes. A. Cup-shaped morphology of purified Mexo (arrowheads) assessed by TEM. B. The particle size and particle concentration of Mexos were analyzed by nanoparticle tracking analysis (NTA). C. Representative images of western blotting showing the exosomal protein markers. D. qRT-PCR results showing let-7b-5p expression levels in arteries of CKD patients (unpaired t-test, n>3). **** $p < 0.0001$, n.s. not significant.

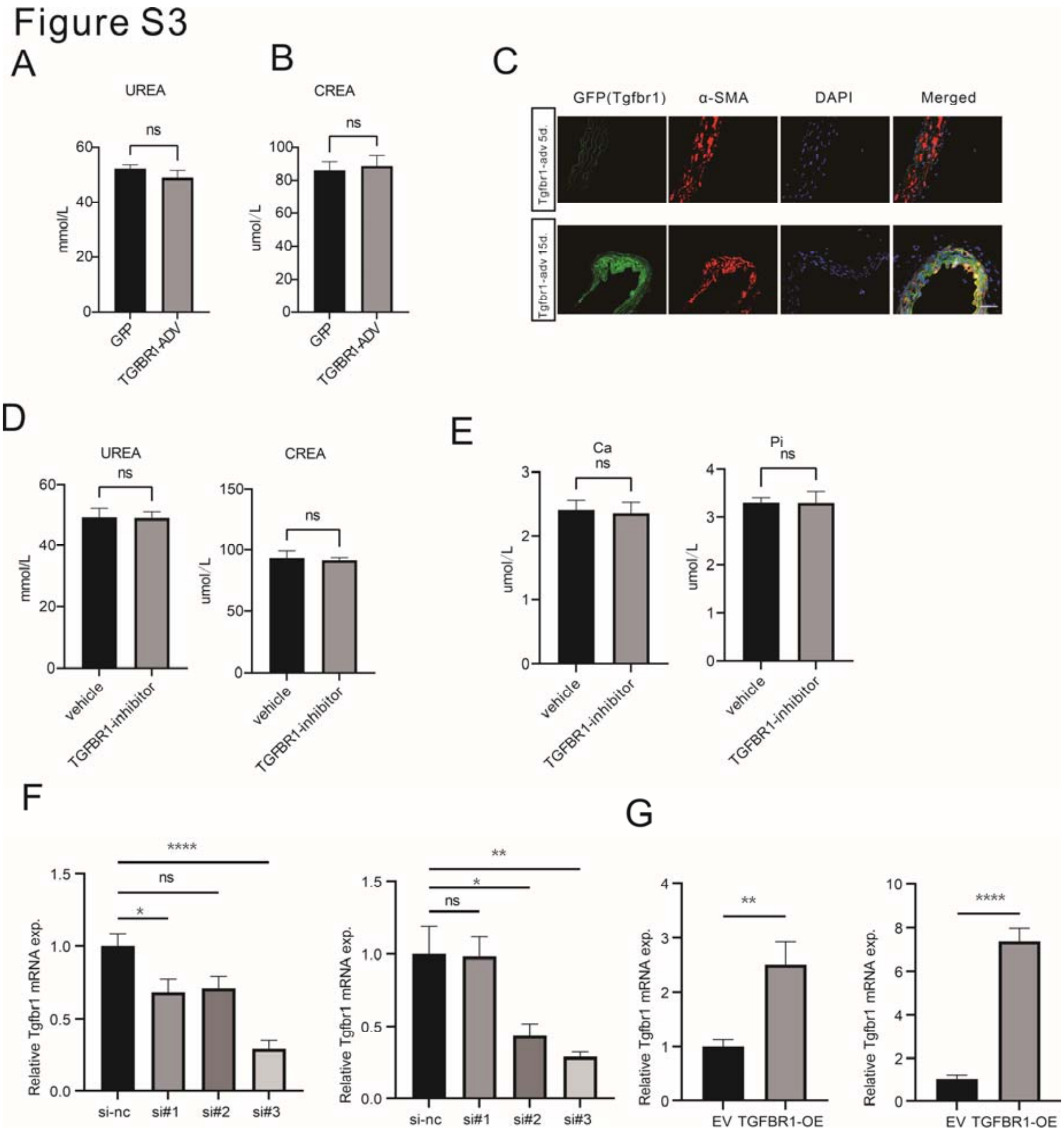

Figure S3. Characteristic of CKD mice with TGFBR1 overexpression or inhibition: blood urea nitrogen (A) and creatine (B) levels in CKD mice treated with Tgfbr1-adv or GFP-adv (unpaired *t*-test, n = 5); representative fluorescence microscopy (C) images of aortas from mice injected with Tgfbr1-adv for 5 days or 15 days (n = 5); (D) blood urea nitrogen (left) and creatine (right) levels in CKD mice treated with Tgfbr1-inhibitor or vehicle (unpaired *t*-test, n = 5); (E) blood calcium (right) and phosphate (left) levels in CKD mice treated with Tgfbr1-inhibitor or vehicle (unpaired *t*-test, n = 5); (F) MOVAS (left) or HASMC (right) cells were transfected with TGFBR1 siRNAs, RNA was extracted after 72 h of culture, and TGFBR1 mRNA levels were detected with qRT-PCR; (G) MOVAS (left) or HASMC (right) cells were transfected with TGFBR1 overexpression plasmids (TGFBR1-OE) or empty vehicle (EV), RNA was extracted after 72 h of culture, and TGFBR1 mRNA levels were detected with qRT-PCR; scale bar, 40 μM. * $p < 0.05$, ** $p < 0.01$, **** $p < 0.0001$, n.s. not significant.
